# Genetic and epigenetic stability of oligodendrogliomas at recurrence

**DOI:** 10.1186/s40478-017-0422-z

**Published:** 2017-03-07

**Authors:** Koki Aihara, Akitake Mukasa, Genta Nagae, Masashi Nomura, Shogo Yamamoto, Hiroki Ueda, Kenji Tatsuno, Junji Shibahara, Miwako Takahashi, Toshimitsu Momose, Shota Tanaka, Shunsaku Takayanagi, Shunsuke Yanagisawa, Takahide Nejo, Satoshi Takahashi, Mayu Omata, Ryohei Otani, Kuniaki Saito, Yoshitaka Narita, Motoo Nagane, Ryo Nishikawa, Keisuke Ueki, Hiroyuki Aburatani, Nobuhito Saito

**Affiliations:** 1 Department of Neurosurgery, Graduate School of Medicine, The University of Tokyo, 7-3-1 Hongo, Bunkyo-ku, Tokyo, 113-8655 Japan; 2 Genome Science Division, Research Center for Advanced Science and Technology, The University of Tokyo, 4-6-1 Komaba, Meguro-ku, Tokyo, 153-8904 Japan; 3 Department of Pathology, Graduate School of Medicine, The University of Tokyo, 7-3-1 Hongo, Bunkyo-ku, Tokyo, 113-8655 Japan; 4 Division of Nuclear Medicine, Department of Radiology, Graduate School of Medicine, The University of Tokyo, 7-3-1 Hongo, Bunkyo-ku, Tokyo, 113-8655 Japan; 5 Department of Neurosurgery and Neuro-Oncology, National Cancer Center Hospital, 5-1-1 Tsukiji, Chuo-ku, Tokyo, 104-0045 Japan; 6 Department of Neurosurgery, Kyorin University Faculty of Medicine, 6-20-2 Shinkawa, Mitaka-City, Tokyo, 181-8611 Japan; 7 Department of Neuro-Oncology/Neurosurgery, Saitama International Medical Center, Saitama Medical University, 1397-1 Yamane, Hidaka-shi, Saitama 350-1298 Japan; 8 Department of Neurosurgery, Dokkyo Medical University, 880 Kitakobayashi, Mibu-machi, Shimotsuga-gun, Tochigi 321-0293 Japan

**Keywords:** Oligodendroglioma, Mutation, Methylation, Heterogeneity, Hypermutator

## Abstract

Among diffuse gliomas, oligodendrogliomas show relatively better prognosis, respond well to radiotherapy and chemotherapy, and seldom progress to very aggressive tumors. To elucidate the genetic and epigenetic background for such behavior and tumor evolution during tumor relapse, we comparatively analyzed 12 pairs of primary and recurrent oligodendrogliomas with 1p/19q-codeletion. Initial treatment for these patients was mostly chemotherapy alone. Temozolomide was used for 3, and procarbazine, nimustine and vincristine (PAV chemotherapy) were used for 7 patients. World Health Organization histological grade at recurrence was mostly stable; it was increased in 2, the same in 9, and decreased in 1 cases. Whole-exome sequencing demonstrated that the rate of shared mutation between the primary and recurrent tumors was relatively low, ranging from 3.2-57.9% (average, 33.3%), indicating a branched evolutionary pattern. The trunk alterations that existed throughout the course were restricted to *IDH1* mutation, 1p/19q-codeletion, and *TERT* promoter mutation, and mutation of the known candidate tumor suppressor genes *CIC* and *FUBP1* were not consistently observed between primary and recurrent tumors. Multiple sampling from different regions within a tumor showed marked intratumoral heterogeneity. Notably, in general, the number of mutations was not significantly different after recurrence, remaining under 100, and no hypermutator phenotype was observed. *FUBP1* mutation, loss of chr. 9p21, and *TCF12* mutation were among a few recurrent *de novo* alterations that were found at recurrence, indicating that these events were clonally selected at recurrence but were not enough to enhance malignancy. Genome-wide methylation status, measured by Illumina 450 K arrays, was stable between recurrence and the primary tumor. In summary, although oligodendroglioma displays marked mutational heterogeneity, histological malignant transformation accompanying events such as considerable increase in mutation number and epigenetic profile change were not observed at recurrence, indicating that noticeable temporal and spatial genetic heterogeneity in oligodendrogliomas does not result in rapid tumor progression.

**Electronic supplementary material:**

The online version of this article (doi:10.1186/s40478-017-0422-z) contains supplementary material, which is available to authorized users.

## Introduction

The recently updated World Health Organization (WHO) classification of central nervous system (CNS) neoplasms incorporated molecular information into the definition of some CNS tumors, thereby officially turning a page into the era of molecular diagnosis of CNS neoplasms. Among such neoplasms, oligodendroglioma was defined as IDH-mutant and 1p/19q-codeleted, making the 1p/19q-codeletion part of the definition of this tumor a quarter of a century after it was first noticed in oligodendroglioma [33]. This genetic alteration is caused by unbalanced translocation of chromosome (chr.) 19p to 1q, leading to the whole arm loss of 1p and 19q. Recent research using next-generation sequencing analysis has revealed the mutational landscape of lower-grade gliomas including oligodendroglioma [13, 35]. Interestingly, the 1p/19q-codeletion has tight positive association with *IDH* mutations and *TERT* promoter mutations, while it is mutually exclusive with *ATRX* loss and *TP53* mutation, which are the hallmark of diffuse astrocytoma, IDH-mutant. Some (30-60%) of 1p/19q-codeleted tumors also have accompanying mutations of *CIC*, *FUBP1* or *NOTCH1*, but these mutations do not appear to be essential for establishment of the histological and clinical features of oligodendrogliomas [4]. Although it is still unknown how 1p/19q-codeletion contributes to the oncogenesis of oligodendroglioma, this alteration is known to be clinically important because tumors with 1p/19q-codeletion have shown remarkable response to combined chemotherapy with procarbazine, lomustine, and vincristine (PCV therapy) [11], which has been confirmed in multiple clinical trials [8, 10, 37]. In contrast to diffuse astrocytoma, IDH-mutants that often undergo malignant progression [3, 20], oligodendroglioma has longer progression free survival and a lower tendency to progress to very aggressive tumors [22]. However, again, the molecular mechanisms that underlie such behaviors are not well known. To gain insight into the molecular mechanism underlying this behavior of oligodendroglioma, we investigated the genetic and epigenetic profile of 1p/19q-codeleted oligodendroglioma at recurrence and compared them to those of the original tumor.

## Materials and methods

### Patients and samples

Tumor samples and paired normal blood samples were obtained at Dokkyo Medical University Hospital, Tokyo University Hospital, National Cancer Center Hospital, and Kyorin University Hospital. Details of the patient characteristics and clinical course are provided in Additional file 1: Table S1. *IDH1/2* mutation was examined by Sanger sequencing, and 1p/19q-codeletion was examined using microsatellite analysis or multiplex ligation-dependent probe amplification (MLPA) methods, spanning the centromeric to telomeric loci to detect the whole arm deletion as described previously [29, 30]. Histological diagnoses were made according to the 2016 WHO guidelines by an experienced neuropathologist in each of the respective treatment centers and were further reviewed by a senior neuropathologist (J. S.). In the recently updated classification, all tumors should be classified as oligodendroglioma or anaplastic oligodendroglioma. This study was approved by the ethics committees of each institute and written informed consent was obtained from all patients.

### DNA and RNA extraction

The AllPrep DNA/RNA Micro kit (Qiagen) was used to extract DNA and RNA from fresh frozen tumor tissue, following the manufacturer’s protocols. The QIAamp DNA Mini Kit (Qiagen) was used to extract control genomic DNA from the paired blood samples. The Qubit Assay Kit (Thermo Fisher Scientific) was used to measure the concentration of double-stranded DNA, and the 2100 Bioanalyzer system (Agilent Technologies) was used to measure the quality of RNA. The RNA Integrity Number (RIN) was > 7 in most of the RNA samples.

### Exome sequencing

Whole exons were enriched using the SureSelect Human All Exon Kit (Agilent) following the manufacturer’s protocols. The capture version is shown in Additional file 1: Table S2. Sequencing was performed as 100-bp pair-ended reads using the HiSeq2000 (Illumina).

### Mutation identification

The Burrows-Wheeler Aligner (BWA) [27] and Novoalign software (Novocraft Technologies) were used to align next-generation sequencing (NGS) reads to the human reference genome GRCh37/hg19. After removal of PCR duplicates, short-read micro re-aligner (SRMA) [18] was used to improve variant discovery through local realignments. To identify somatic mutations, we used an integrated genotyper software (karkinos: http://sourceforge.net/projects/karkinos/) that detects single nucleotide variants (SNVs), copy number variation (CNV) and tumor purity [21]. A heuristic algorithm was used for SNV detection as previously reported [21, 36]. Somatic mutant allele frequencies adjusted by estimated tumor content ratios that were ≥15% were retained. Artifacts originating from errors in the sequence and mapping were also filtered by heuristic filtering and Fisher’s test. To eliminate germline variations in this study, we carried out comparative analyses using paired tumor and normal samples for each of the samples analyzed and we extracted the somatic events detected only in tumor tissues. Mutations were validated by Sanger sequencing or by RNA sequencing.

### *TERT* promoter mutation

Mutations within the *TERT* promoter regions were detected by Sanger sequencing with previously reported primers [23].

### Copy number analysis

Read depths were compared between normal and tumor for each capture target region. After normalizing by number of total reads and GC content bias, the tumor/normal depth ratio was calculated and values were smoothed using a moving average. Copy number peaks were then estimated using wavelet analysis, and each peak was approximated using complex Gaussian models. A hidden Markov model (HMM) and calculated Gaussian models were constructed and copy number peaks were linked to a genomic region. Allelic imbalance for each copy number peak was then calculated, and imbalance information and peak distance were further analyzed by model fitting, yielding integer copy number annotation and tumor purity [21].

### RNA sequencing

An RNA sequencing library was prepared using the TruSeq Stranded mRNA LT Sample Prep Kit (Illumina) according to the manufacturer’s protocol. Briefly, 1 μg of total RNA was purified using oligo dT magnetic beads and poly A+ RNA was fragmented by heating at 94 °C for 2 min. cDNA was synthesized using SuperScript II (Invitrogen) and adapter ligated cDNA was amplified using 12 cycles of PCR. Each library was sequenced using HiSeq2000, in which four libraries were loaded per lane of the flowcell, producing an average of 28.7 million pairs of 101-cycle reads for each sample.

NGS reads were independently mapped to a cDNA database (UCSC genes) and a reference genome (GRCh37/hg19) using the BWA. After a cDNA coordinate was converted to genomic positions, the optimal mapping result was chosen either from cDNA or genome mapping by comparing the minimal edit distance to the reference. Subsequently, local realignment was done with an in-house short reads aligner with a small seed size (k = 11) (Qgram-SmithWaterman).

### Genome-wide methylation profiling

The Infinium assay was performed according to Illumina’s standard protocol using the Infinium Human Methylation 450 K BeadChip (Illumina). For each CpG site, the β-value was calculated by using the following equation: intensity of the Methylated allele (M) / intensity of the Unmethylated allele (U) + intensity of the Methylated allele (M) +100 [5]. This β-value, which ranged from 0 (unmethylated) to 1 (fully methylated), reflects the methylation level of the individual CpG site represented by the probe.

We downloaded Infinium Human Methylation 450 K BeadChip data of 289 lower-grade gliomas from The Cancer Genome Atlas (TCGA) Data Portal (https://tcga-data.nci.nih.gov/docs/publications/lgg_2015) and analyzed these data together with the data of our 30 samples. The following filtering steps were used to select probes for unsupervised clustering analysis. Probes targeting the X and Y chromosomes, and probes associated with a single nucleotide polymorphism (SNP) according to TCGA data, [7, 13] were excluded. The standard deviation (SD) of β-values for each probe was calculated and the top 8000 probes were selected.

### Promoter methylation of the O^6^-methylguanine DNA methyltransferase (MGMT) gene

We adopted the MGMT-STP27 model, using two Infinium Human Methylation 450 K BeadChip probes cg12434587 and cg12981137 [2], to determine *MGMT* promoter methylation status.

## Results

### Characteristics of oligodendroglioma cases

We sequenced 12 pairs of primary and recurrent 1p/19q-codeleted tumors (Additional file 1: Table S1). Of these tumors, 2 progressed from WHO grade II to grade III histologically, 9 remained as the same grade (8 as grade III, 1 as grade II), and 1 grade III tumors were diagnosed as grade II at recurrence (Additional file 2: Figure S1). Regarding the treatment of the primary tumors, all but one patient was treated with chemotherapy; 3 patients were treated with temozolomide (TMZ), 7 patients with a combination of procarbazine, nimustine (ACNU) and vincristine (PAV chemotherapy), and 1 patient with ACNU and vincristine. PAV chemotherapy is commonly used in place of PCV chemotherapy because of the unavailability of lomustine (CCNU) as an approved drug in Japan. In this cohort, four to eight courses (average, 6.9 courses) of PAV therapy were administered. Each course of PAV chemotherapy consisted of procarbazine (at a dose of 100 mg on days 8 through 21), ACNU (at a dose of 80 mg per square meter of body-surface area, administered intravenously on day 1), and vincristine (at a dose of 1.4 mg per square meter administered intravenously on days 1 and 29). The cycle length was 6-8 weeks. Drug dosage was reduced when a severe side effect was observed in the prior course. In addition to chemotherapy, 2 patients also received radiation therapy, and the one remaining patient was treated with radiation therapy alone. The diagnosis of recurrence and the subsequent decision to proceed with resection were made when the emergence of a gadolinium-enhanced lesion (3 cases) or a noticeable enlargement of a FLAIR-high region (9 cases) was observed on MRI. Methionine PET high uptake of the recurrent lesion was also used as supportive data to make a diagnosis of recurrence in 9 cases. These 12 patients were followed up for a median time of 52 months after the second surgery, and 8 patients were still alive at that time, indicating that most of the recurrent oligodendrogliomas could still be controlled by the treatment used (Additional file 1: Table S1).

In addition, to investigate intratumoral heterogeneity, in 4 cases we analyzed paired samples obtained from two different regions that showed different imaging features within the tumor. The imaging study used to decide these regions was ^11^C-methionine positron emission tomography (MET-PET) in 3 cases, and Gd-enhanced MRI in 1 case. A computerized navigation system was used to obtain the samples from targeted lesions. Histological diagnosis of all paired samples was determined independently based on the H/E-stained slides made from the corresponding tissues (Additional file 1: Table S1 and Additional file 2: Figure S2). All 4 patients were still alive during follow-up ranging from 42 – 54 (average 47) months after surgery. Only one patient (patient 14) showed local tumor recurrence.

### Mutation analysis

**Fig. 1:**
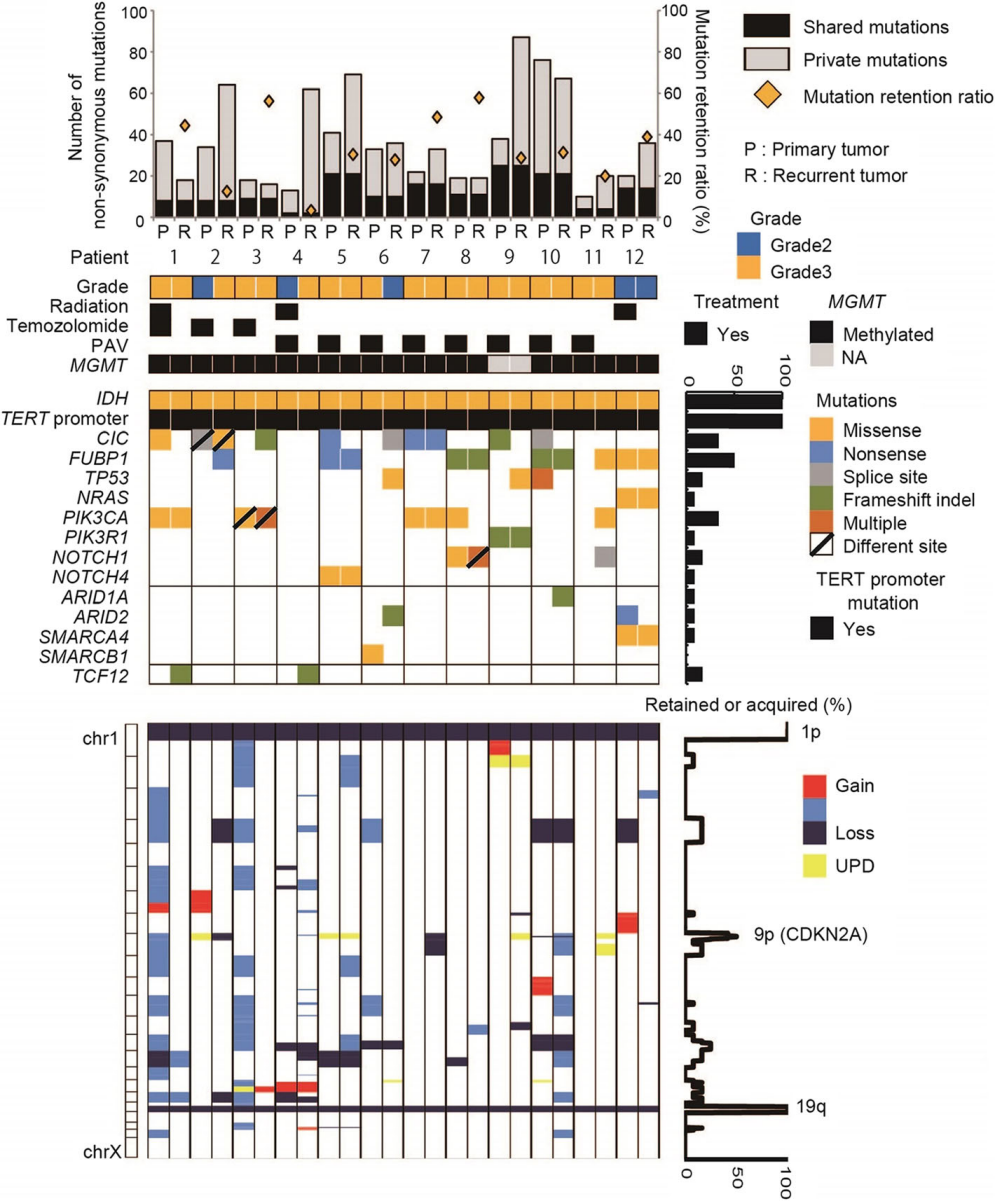
Summary of the genomic profiles of the primary and recurrent oligodendrogliomas. The number of non-synonymous mutations, WHO grade, postoperative treatment, *MGMT* promoter methylation status, mutation profiles and copy number alterations are shown from top to bottom of the panel. On the right of the panel, the percentage of retained and acquired mutations and copy number alterations are depicted

We performed exome sequencing of 32 tumors and 16 matched normal blood samples. The sequencing data are summarized in Additional file 1: Table S2. Sequencing depths for tumors and normal blood samples were 112.8× and 105.5× on averages, respectively, meaning that sufficient numbers of reads for mutant alleles could be obtained. At a sequencing depth of more than X20, 95.2 ± 2.0% of the coding sequence was covered on average. The estimated tumor purity was 78.9 ± 17.8% (ranging from 41.3 - 98.0%) on average. In total, we detected 1138 non-synonymous mutations in 32 oligodendroglioma samples analyzed by exome sequencing. A list of the mutations and detailed information are shown in Additional file 1: Table S3. For validation, 100 mutations were randomly selected from samples with matched RNA sequencing data. Of these 100 mutations, 41 had more than 10 reads in RNA sequencing, and at least 34 of 41 (83%) mutations were confirmed, indicating reasonably high reliability of the exome sequencing. The average number of non-synonymous mutations in 12 pairs of primary and recurrent tumors was 30 (10 to 76) and 43 (16 to 87), respectively (Fig. 1). The retention rate, i.e., the fraction of initial tumor mutations preserved in the recurrent tumor [24], was 33.3% on average (3.2 to 57.9), which demonstrated that mutational similarity of primary and recurrent tumors was low in general, but varied significantly among the cases. Hypermutated tumors, which have been reported in recurrence of glioblastomas and astrocytomas [12, 20] were not found in the present recurrent oligodendrogliomas, even after 12 courses of TMZ chemotherapy. Two cases that demonstrated progression at recurrence had the lowest retention rates of 3.2% (patient 4) and 12.5% (patient 2). A different mutation in the same gene was observed in primary and recurrent tumors that involved *CIC* in patient 2 and *PIK3CA* in patient 3, which demonstrated a convergent evolution pattern. To deduce the degree of advantage provided by each mutation for tumor growth, we focused on mutations that were retained or acquired in recurrent tumors. In some cases, even well-known presumptive major driver mutations of *CIC*, *TP53* and *PIK3CA* that were detected in primary tumors were not detected at recurrence (mutations in *CIC* in patient 1, 5, 9 and 10; in *TP53* in patient 10; and in *PIK3CA* in patient 8). *FUBP1* mutation was seen in six out of the twelve recurrent tumors; such mutation was acquired in recurrent tumors in 2 cases (patients 2 and 11), and the same *FUBP1* mutation as that in the primary tumor was retained in the recurrent tumor in 4 cases (patients 5, 8, 10, and 12). There was no case in which *FUBP1* mutation was lost at recurrence. *TCF12* mutation was acquired in two out of the twelve recurrent tumors (patients 1 and 4).

### Copy number aberrations

**Fig. 2:**
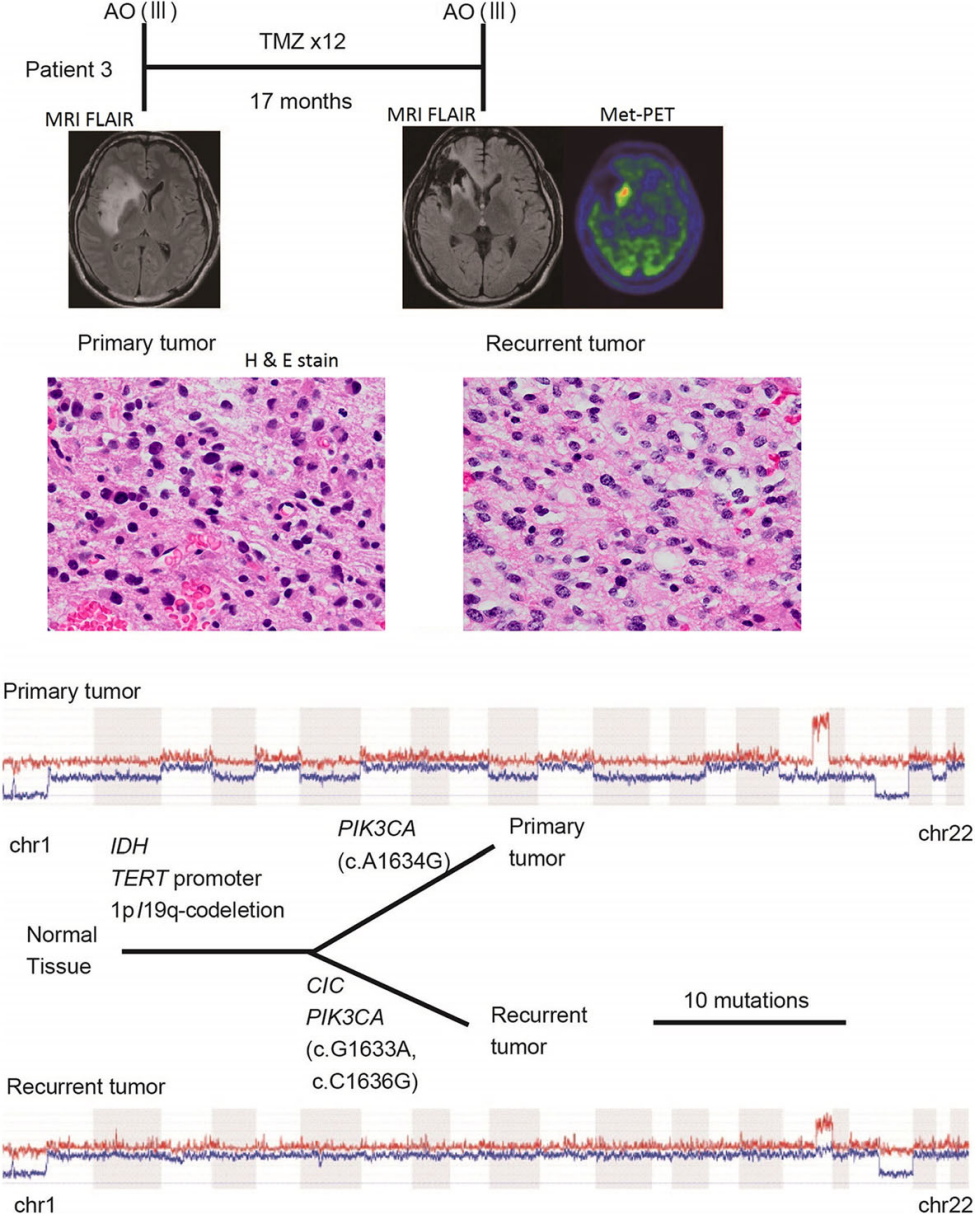
An example of histopathological and molecular genetic alterations in recurrent anaplastic oligodendroglioma (Patient 3). The patient with anaplastic oligodendroglioma was treated with 12 courses of temozolomide after gross total removal of the tumor, and MRI and ^11^C-Methionine –PET detected regrowth of the tumor 17 months after this treatment. Histological analysis was anaplastic oligodendroglioma at recurrence, which showed decreased tumor cell density, slight improvement of atypia of the nucleus, and decreased number of mitotic cells compared to the primary tumor. Hemizygous loss of multiple foci was not preserved at recurrence, except for 1p/19q-codeletion. Clinical course, MRI and MET-PET images, pathological images (H&E stained formalin-fixed paraffin-embedded tissue slides), copy number alterations and phylogenetic tree of the primary and recurrent tumor are depicted from top to bottom of the panel

Chromosome 1p/19q-codeletion was retained in all recurrent tumors. Frequent copy number alteration (CNA) was observed at the 9p21 locus containing the *CDKN2A* gene, where allelic loss or uniparental disomy (UPD) was observed in 8 cases. In our series, an increase in genomic instability was not typical at recurrence and some recurrent tumors even demonstrated decreased genomic aberrations compared to those of the respective primary tumors. For example, in patient 3 (Fig. 2), the primary tumor was anaplastic oligodendroglioma and, after 12 courses of TMZ chemotherapy, the tumor recurred along the margin of the resection cavity. The recurrent tumor showed noticeable enlargement of a high intensity region on MRI fluid-attenuated inversion recovery (FLAIR) image with high uptake by MET-PET, and was surgically resected. The pathological diagnosis was still anaplastic oligodendroglioma; however, tumor cells were sparse, atypia of the nucleus was slightly improved, and the number of mitotic cells was decreased in the recurrent tumor compared to the primary tumor. Both the primary and the recurrent tumors had mutations in *IDH1* and the *TERT* promoter as well as 1p/19q-codeletion. *PIK3CA* mutations were also commonly found, although the mutated position in the gene was different between the recurrent and the primary tumor, indicating that convergent evolution had occurred in the course of tumor growth. *CIC* mutation was found only in the recurrent tumor. In that case, chromosomal instability, which may be related to the formation of malignancy, was evident only in the primary tumor. This patient is still healthy 76 month after the second surgery.

### Intratumoral heterogeneity

**Fig. 3:**
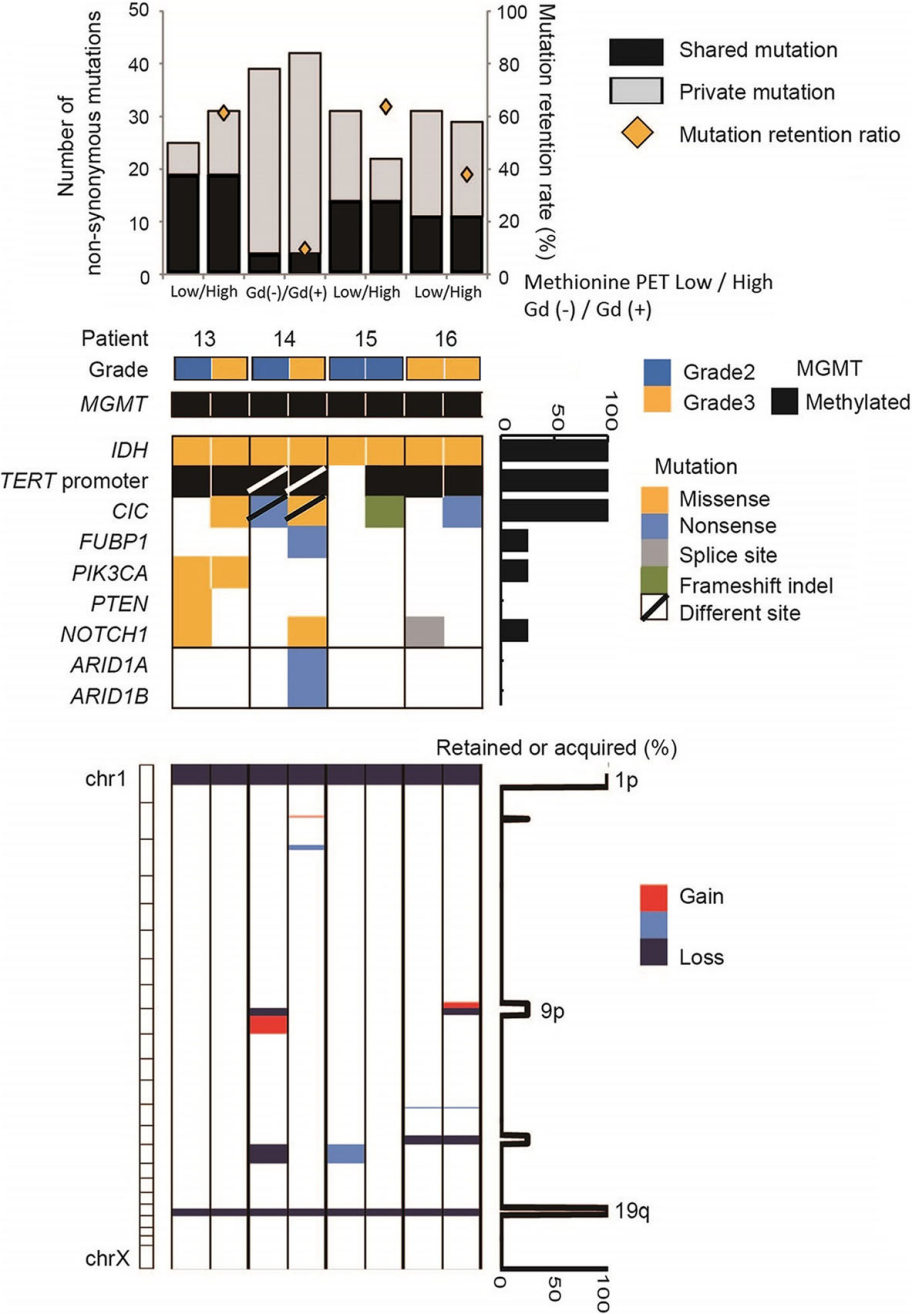
Summary of genomic profiles in different regions of oligodendrogliomas. Mutation and copy number analysis of samples in different regions of the tumor in four patients. The number of non-synonymous mutations, clinical grade, *MGMT* promoter methylation status, mutation profiles and copy number alterations are shown from top to bottom of the panel. On the right of the panel, the percentage of retained and acquired mutations and copy number alterations are depicted

**Fig. 4:**
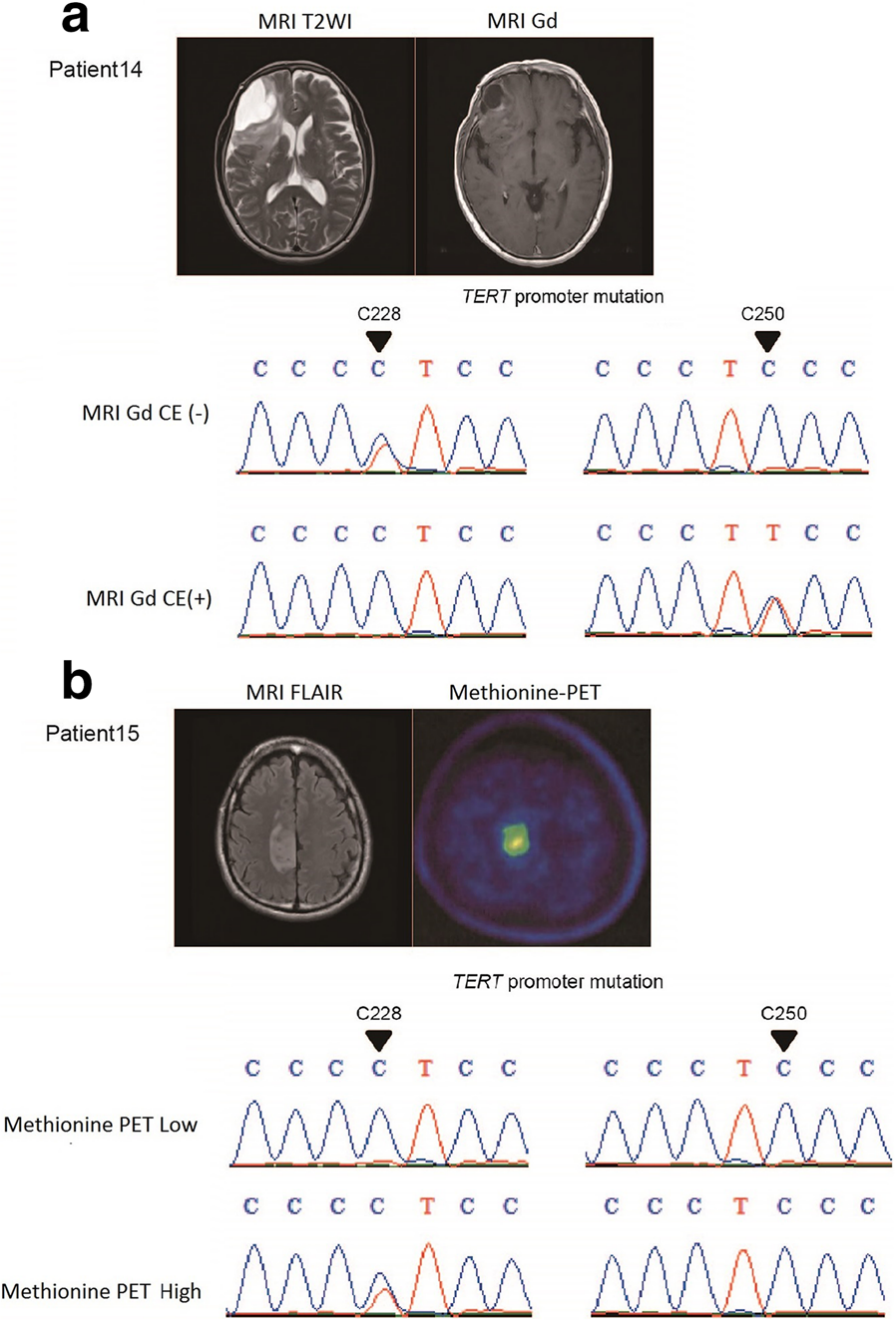
Representative cases illustrating intratumoral heterogeneity. **a** In patient 14, the Gd-enhanced tumor center and the marginal non-enhanced part of the tumor were separately collected. The *TERT* promoter mutation was different in each tumor region. **b** In patient 15, the tumor center with MET-PET high uptake, and the marginal tumor portion with MET-PET low uptake, were separately collected. Only the tissue with MET-PET high uptake had *TERT* promoter mutation

In four cases, we were able to analyze multiple samples obtained from different tumor regions, where a difference in methionine uptake on MET-PET or in enhancement by gadolinium on MRI was observed preoperatively in the same tumor. The rate of shared mutation was 43.1% on average (9.5 - 63.6%). Similar to the analysis comparing primary and recurrent tumors, the mutation status differed among tumor regions, even that of major driver genes such as *CIC, FUBP1, PTEN,* and *NOTCH1* (Fig. 3). *IDH1* mutations were identical in both tumor regions in all 4 cases. Of note, however, different *TERT* promoter mutation status between regions was observed in two samples; in case 14, one Gd non-enhancing portion had a C228T mutation and the other enhancing portion had a C250T mutation; in patient 15, only the histologically progressive portion had a C228T mutation (Fig. 4). *CIC* mutations were identified in all 4 tumor regions with high methionine uptake or enhancement, while no *CIC* mutation was found in regions with low methionine uptake or no Gd enhancement. In copy number analysis, 2 cases showed a difference in 9p status, and 2 cases showed a difference in chr. 15 status between regions, while the 1p/19q-codeletion was present in all tumor regions.

### Methylation analysis

**Fig. 5:**
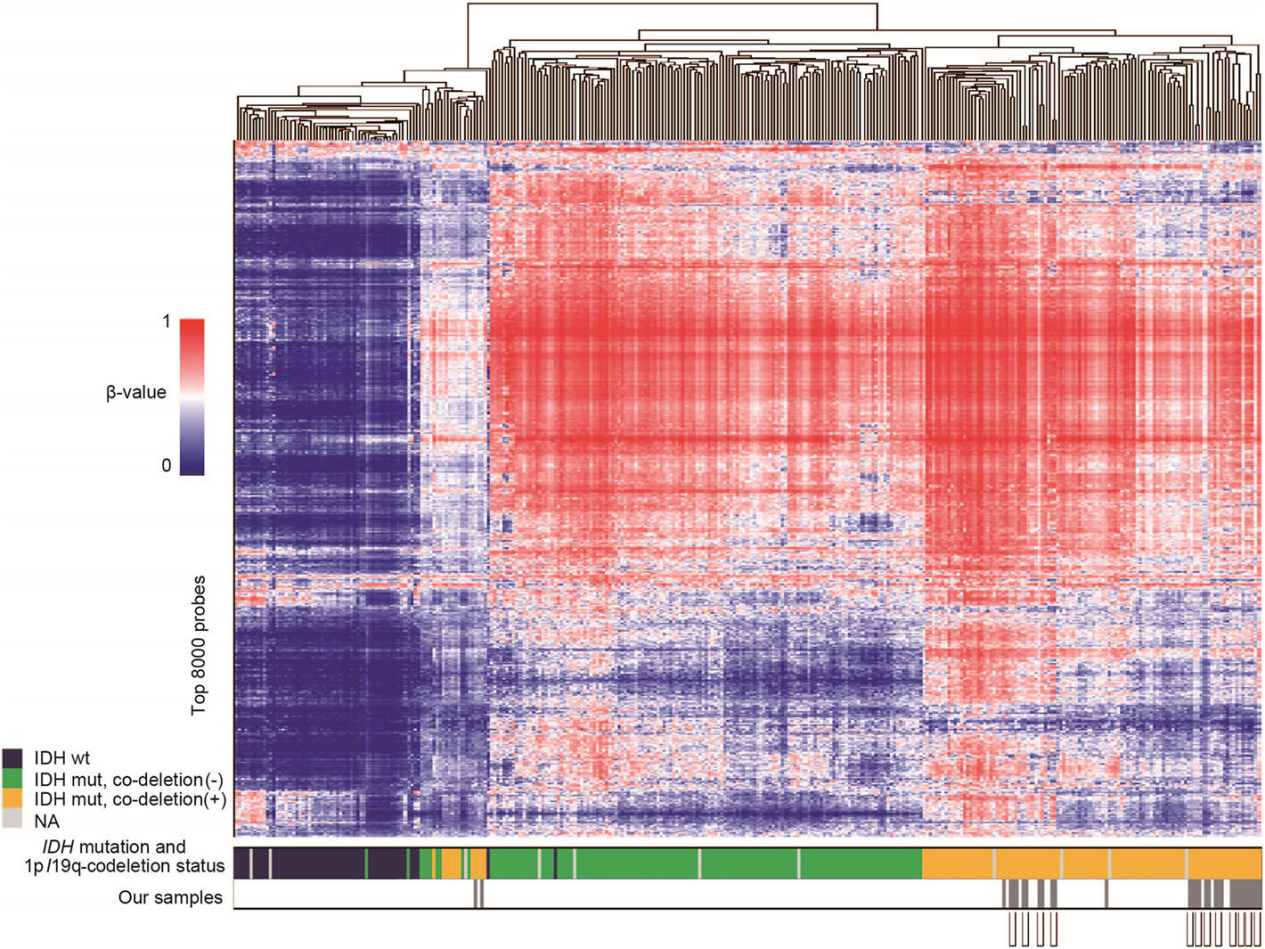
Genome-wide methylation status was stable between recurrence and the primary tumor. Heatmap of the methylation levels (β-value) in 319 samples, including the 30 oligodendrogliomas of the present study and 289 lower-grade gliomas from TCGA. Unsupervised clustering was performed using 8000 selected Infinium probes. Each row represents a probe, and each column represents one sample. For each sample, *IDH1* mutation and 1p/19q-codeletion status are indicated by colored boxes at the bottom of the map. Samples connected with a line are pairs of primary and recurrent tumors or samples in different regions of the tumor

We performed unsupervised clustering with 319 samples, which were comprised of the 30 oligodendrogliomas of the present study and 289 lower-grade gliomas from TCGA (Fig. 5). Except for three cases, of which two seemed to have a low tumor content in one of the pair, paired samples were clustered next to each other, and robust methylation change at recurrence or in different regions within each tumor was not observed; i.e., when primary 1p/19q-codeleted tumors were in the cluster with higher methylation, the recurrent tumors stayed in the same cluster. When primary tumors were in the less methylated cluster, recurrent tumors also stayed in the same cluster.

The *MGMT* promoter region was methylated in all tumors (Figs. 1 and 3), indicating that *MGMT* promoter methylation had not changed at recurrence or in different regions within the same tumor.

## Discussion

In the present study, similar to the results of previous studies of astrocytic tumors [3, 20, 35], the mutation retention rate at recurrence in oligodendrogliomas was relatively low. In ten out of the twelve tumors, more than half of the mutations found in the primary tumor were not found in the recurrent tumor. These observations indicated that oligodendrogliomas show a complex branched evolutionary pattern at recurrence similar to other malignant gliomas. Indeed, in some recurrent tumors, mutations such as *CIC*, *TP53*, and *PIK3CA* mutations that are generally regarded as potent drivers were not maintained at recurrence. On the other hand, mutations in *FUBP1*, which is a transcriptional modulator of c-MYC [19], were maintained or newly acquired at recurrence, suggesting that these *FUBP1* mutations may confer a survival advantage. Similarly, although less frequently, inactivating *TCF12* mutations were acquired in 2 recurrent tumors; these mutations were frameshift indels, p.97_97del in patient 1 and p.I162fs in patient 4. Mutations leading to truncation of a basic helix-loop-helix (bHLH) domain of the transcription factor TCF12 were previously detected in an aggressive type of 1p/19q-codeleted tumor [26]. Those results, together with our data, mean that such truncation can be considered as one of the driving genetic alterations in recurrent oligodendroglioma. Regarding copy number alterations, apart from 1p/19q-codeletion, the 9p21 locus containing the *CDKN2A* gene was the most frequently altered locus. Alteration of this locus was not so frequent in 1p/19q-codeleted tumors in previous large scale analyses [13, 35]. However, alteration of the 9p21 locus was previously reported to be associated with histological malignancy such as microvascular proliferation and necrosis [6, 15], as well as worse prognosis in 1p/19q-codeleted tumors [1]. Even though the alterations described above might have been clonally selected at recurrence and were potentially associated with tumor growth, there was no increase in malignant histological characteristics in most of the recurrent tumors, and most of such tumors could still be controlled by the treatment, demonstrating that these events were not sufficient to enhance tumor malignancy.

The rise of a hypermutator phenotype after TMZ chemotherapy against low-grade gliomas has been reported, raising some concern regarding the management strategy for this tumor [20]. In our series of 12 pairs of primary and recurrent tumors, in which PAV chemotherapy was used in the majority (7/12), neither a significant increase in mutation number in recurrent tumors nor a hypermutator phenotype was observed. One possible reason for the absence of hypermutation in our series is that astrocytic tumors with *IDH* mutation may be more prone to a hypermutator phenotype compared to oligodendrogliomas, since a previous report demonstrated that a hypermutator phenotype is frequently found in astrocytic tumors harboring *IDH* mutation that were treated with TMZ [20, 24]. It has also been reported that a hypermutator phenotype might be infrequent in glioblastoma without *IDH* mutation, suggesting that the incidence rate of the hypermutator phenotype is different among glioma subtypes [24]. Another possible explanation is that only three cases were treated with TMZ, which is thought to be a major driver for the hypermutator phenotype, and that PAV therapy, which is an analogous regimen to PCV chemotherapy in Japan, is less likely to cause a hypermutator phenotype. The emergence of a hypermutator phenotype is thought to be related to the mechanism of action of TMZ, and to chemical reactions of alkylating agents belonging to the triazene group such as TMZ, procarbazine, and dacarbazine, which differ from those of nitrosoureas such as ACNU, BCNU, and CCNU. Briefly, TMZ adds a methyl group to the O^6^ position of a guanine residue to make O^6^-methylguanine, which leads to the addition of a thymine residue instead of a cytosine into the paired DNA strand when DNA replicates. These mismatch residues are recognized by the mismatch repair system. An attempt to repair this mismatch is then initiated, which cannot be completed in the presence of O^6^-methylguanine and therefore the process ends up with thymine reinsertion, leading to a futile mismatch repair cycle and eventually apoptosis [16, 32]. A defect in the mismatch repair system confers resistance to TMZ but leads to a large amount of C > T/G > A mutations [12, 20]. On the other hand, nitrosoureas such as ACNU add a chloroethyl group to the O^6^ position of a guanine residue, making O^6^-chloroethylguanine, and subsequent cross-linking prevents DNA replication and induces apoptosis [34]. Thus, the mechanism of action of ACNU is not related to the mismatch repair system, and therefore these drugs will not cause a hypermutator phenotype. Although procarbazine that is used in PAV chemotherapy has a similar pharmacological action to TMZ, the dosage and duration of procarbazine treatment are different from those of TMZ and such differences might affect the incidence rate of a hypermutator phenotype. Indeed, there did not seem to be a frequent rise in a hypermutator phenotype after chemotherapy that consisted mainly of nitrosourea in our oligodendroglioma cases. A recent phase III study showed that radiation plus PCV chemotherapy elongates progression-free survival and overall survival of high-risk low-grade glioma, especially of oligodendroglioma [8]. Although TMZ is a candidate substitute for PCV therapy, which often results in relatively severe side effects, it may be necessary to consider such possible different consequences of these regimens and to investigate genomic status in a larger series of recurrent gliomas in the future. Understanding those molecular dynamic features might be essential for planning of the future treatment strategy, including molecular targeting therapy, for sufficient control of this tumor.

Recent studies using whole-exome sequencing have revealed that lower-grade gliomas also harbor intratumoral heterogeneity, which is widely observed in malignant tumors such as glioblastomas [17, 25, 35]. We observed here that oligodendroglioma, which generally has a better prognosis compared with astrocytic tumors, also demonstrates marked intratumoral heterogeneity. Mutant allele frequencies were generally low; < 0.75, < 0.5, and < 0.25 in 96%, 87%, and 42% of the number of identified gene mutations, respectively, which most likely reflect heterogeneity within the tumor. Among genetic and chromosomal alterations, *IDH1* mutation and 1p/19q-codeletion were prevalent in almost all tumor cells and are considered to be the trunk alterations. However, in two analyzed cases, analysis of regions that showed different histological and imaging features within a tumor demonstrated that *TERT* promoter mutation was not identical in these regions. Previous reports also demonstrated slightly less than 100% incidence of *TERT* promoter mutation in 1p/19q-codeleted oligodendrogliomas [13, 35]. In addition, the presence of other mutations that are frequently observed in oligodendrogliomas can vary temporally and spatially within a tumor. Therefore, mutations other than *IDH1* and 1p/19q-codeletion including *TERT* promoter mutation appear to be later events in oligodendroglioma oncogenesis.

The combined data presented here suggest interesting genetic features of oligodendrogliomas. The number of mutations harbored by oligodendroglioma is not necessarily smaller than that reported for astrocytic tumors, and there appears to be marked intratumoral heterogeneity. Although tumor heterogeneity is often related to the malignant nature of tumors, in recurrent oligodendrogliomas there was no tendency for mutation increase at recurrence, and, in some cases, there were fewer mutations and copy number aberrations at recurrence than in the primary tumor. In addition to such observations of genetic and genomic changes, minimal epigenetic changes were observed in recurrent oligodendroglioma compared to the primary tumor. Such epigenetic stability is in contrast to that of astrocytic tumors that harbor *IDH* mutation, which demonstrate a dynamic methylation profile change during malignant progression [14, 28]. In our series, *MGMT* promoter methylation was also stable among tissues from the same patient, although a previous study suggested that there is clonal heterogeneity of *MGMT* promoter methylation even in oligodendroglioma [31].

## Conclusions

Although oligodendroglioma display remarkable spatial and temporal heterogeneity that is known to be related to tumor evolution [9], our study demonstrated that their tumor malignancies are rarely promoted by additionally acquired mutations or genomic aberrations at recurrence. Such molecular characteristics may account for clinically benign nature of oligodendroglioma compared to other diffuse gliomas, and may influence tailored therapeutic strategy for this tumor in the future.

## Additional files

Additional file 1: Table S1. Clinical characteristics of the patient cohort. **Table S2.** Sequence data summary. **Table S3.** List of the somatic mutations in this study. (XLSX 163 kb)

Additional file 2: Figure S1. Histopathological features of primary and recurrent tumors in a 34 year-old female (patient 6). The primary tumor was diagnosed as anaplastic oligodendroglioma (WHO grade III) (A). Postoperatively, the patient was treated with 8 courses of PAV chemotherapy. Eight years after the initial surgery, an MRI FLAIR-high lesion was noticeably enlarged and this region showed high uptake in Methionine PET. Tumor recurrence was therefore suspected and surgical resection was performed. In the recurrent tumor, atypia of the nucleus was improved and numbers of mitotic cells were decreased compared to the primary tumor, and the tumor was diagnosed as oligodendroglioma (WHO grade II) (B). Formalin-fixed paraffin-embedded tissues were sectioned and stained with Hematoxylin and Eosin (bar = 100 μm). **Figure S2.** Histopathological features of different tumor portions from the same patient as listed in Additional file 1: Table S1. Formalin-fixed paraffin-embedded tissues were sectioned and stained with Hematoxylin and Eosin (bar = 100 μm). A. Patient 13, Methionine PET low uptake, grade II; B. Patient 13, Methionine PET high uptake, grade III; C. Patient 14, Gadolinium enhanced -, grade II; D. Patient 14, Gadolinium enhanced +, grade III; E. Patient 15, Methionine PET low uptake, grade II; F. Patient 15, Methionine PET high uptake, grade II; G. Patient 16, Methionine PET low uptake, grade III; H. Patient 16, Methionine PET high uptake, grade III. (PPTX 1597 kb)

## Abbreviations

ACNU: nimusutine
bHLH: basic helix-loop-helix
BWA: Burrows-Wheeler Aligner
CCNU: lomustine
Chr.: chromosome
CNA: copy number alteration
CNS: central nervous system
CNV: copy number variation
FLAIR: fluid-attenuated inversion recovery
HMM: hidden Markov model
MET-PET: ^11^C-methionine positron emission tomography
MGMT: O^6^-methylguanine DNA methyltransferase
MLPA: multiplex ligation-dependent probe amplification
NGS: next-generation sequencing
PAV: procarbazine, nimustine and vincristine
PCV: procarbazine, lomustine and vincristine
RIN: RNA Integrity Number
SD: standard deviation
SNP: single nucleotide polymorphism
SNV: single nucleotide variant
SRMA: short-read micro re-aligner
TCGA: The Cancer Genome Atlas
TMZ: temozolomide
UPD: uniparental disomy
WHO: World Health Organization
