# Supplementary material for: Genetic and epigenetic stability of oligodendrogliomas at recurrence
Source: Acta Neuropathol Commun. 2017 Mar 7;5:18. doi: 10.1186/s40478-017-0422-z (PMC5339990; doi:10.1186/s40478-017-0422-z)
Supplement: Supplementary file 2 — Histopathological features of primary and recurrent tumors in a 34 year-old female (patient 6). The primary tumor was diagnosed as anaplastic oligodendroglioma (WHO grade III) (A). Postoperatively, the patient was treated with 8 courses of PAV chemotherapy. Eight years after the initial surgery, an MRI FLAIR-high lesion was noticeably enlarged and this region showed high uptake in Methionine PET. Tumor recurrence was therefore suspected and surgical resection was performed. In the recurrent tumor, atypia of the nucleus was improved and numbers of mitotic cells were decreased compared to the primary tumor, and the tumor was diagnosed as oligodendroglioma (WHO grade II) (B). Formalin-fixed paraffin-embedded tissues were sectioned and stained with Hematoxylin and Eosin (bar = 100 μm). Figure S2. Histopathological features of different tumor portions from the same patient as listed in Additional file 1: Table S1. Formalin-fixed paraffin-embedded tissues were sectioned and stained with Hematoxylin and Eosin (bar = 100 μm). A. Patient 13, Methionine PET low uptake, grade II; B. Patient 13, Methionine PET high uptake, grade III; C. Patient 14, Gadolinium enhanced -, grade II; D. Patient 14, Gadolinium enhanced +, grade III; E. Patient 15, Methionine PET low uptake, grade II; F. Patient 15, Methionine PET high uptake, grade II; G. Patient 16, Methionine PET low uptake, grade III; H. Patient 16, Methionine PET high uptake, grade III. (PPTX 1597 kb) [file 40478_2017_422_MOESM2_ESM.pptx]

## Slide 1
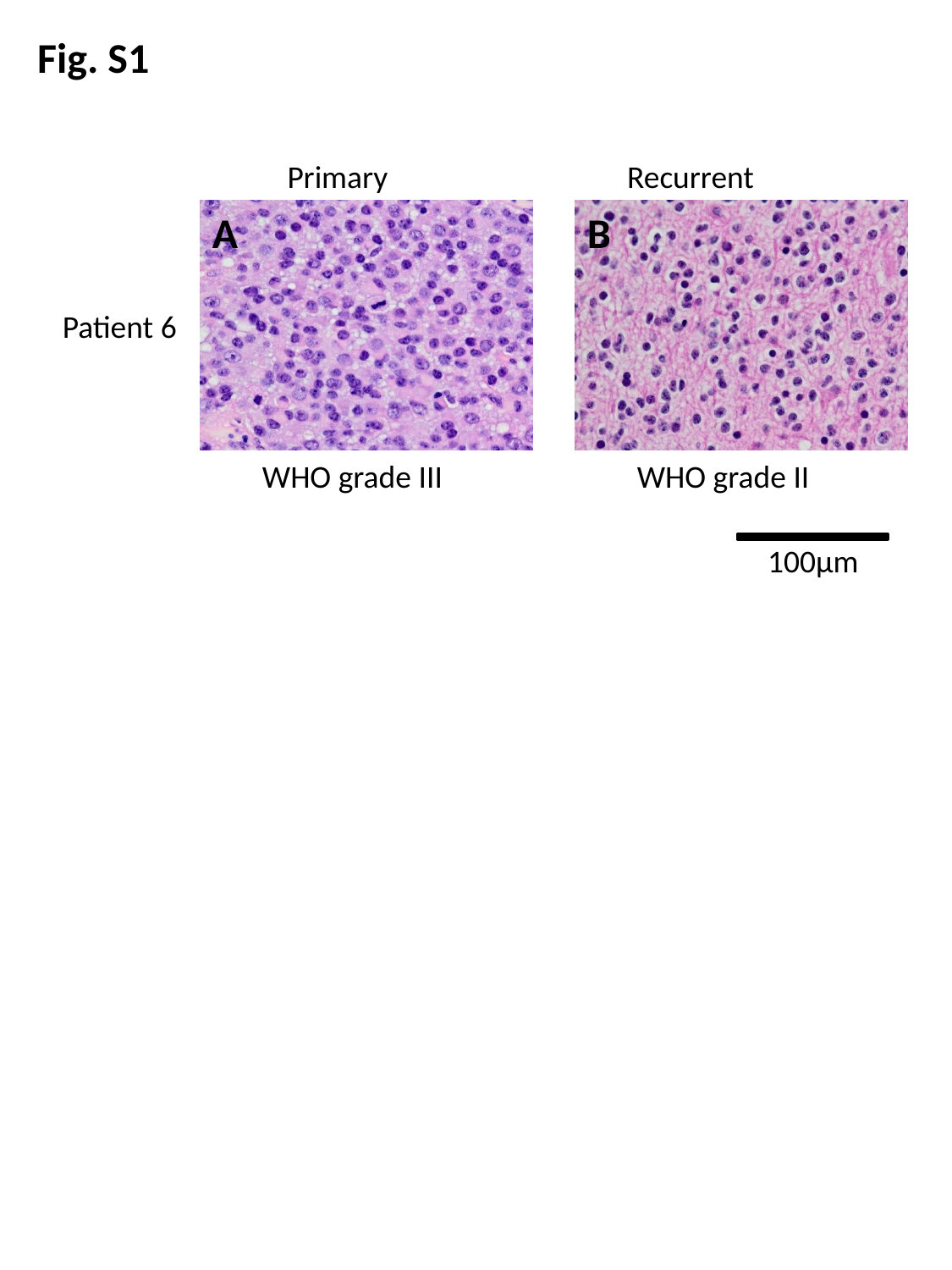

Fig. S1
Primary
Recurrent
A
B
Patient 6
WHO grade III
WHO grade II
100μm

## Slide 2
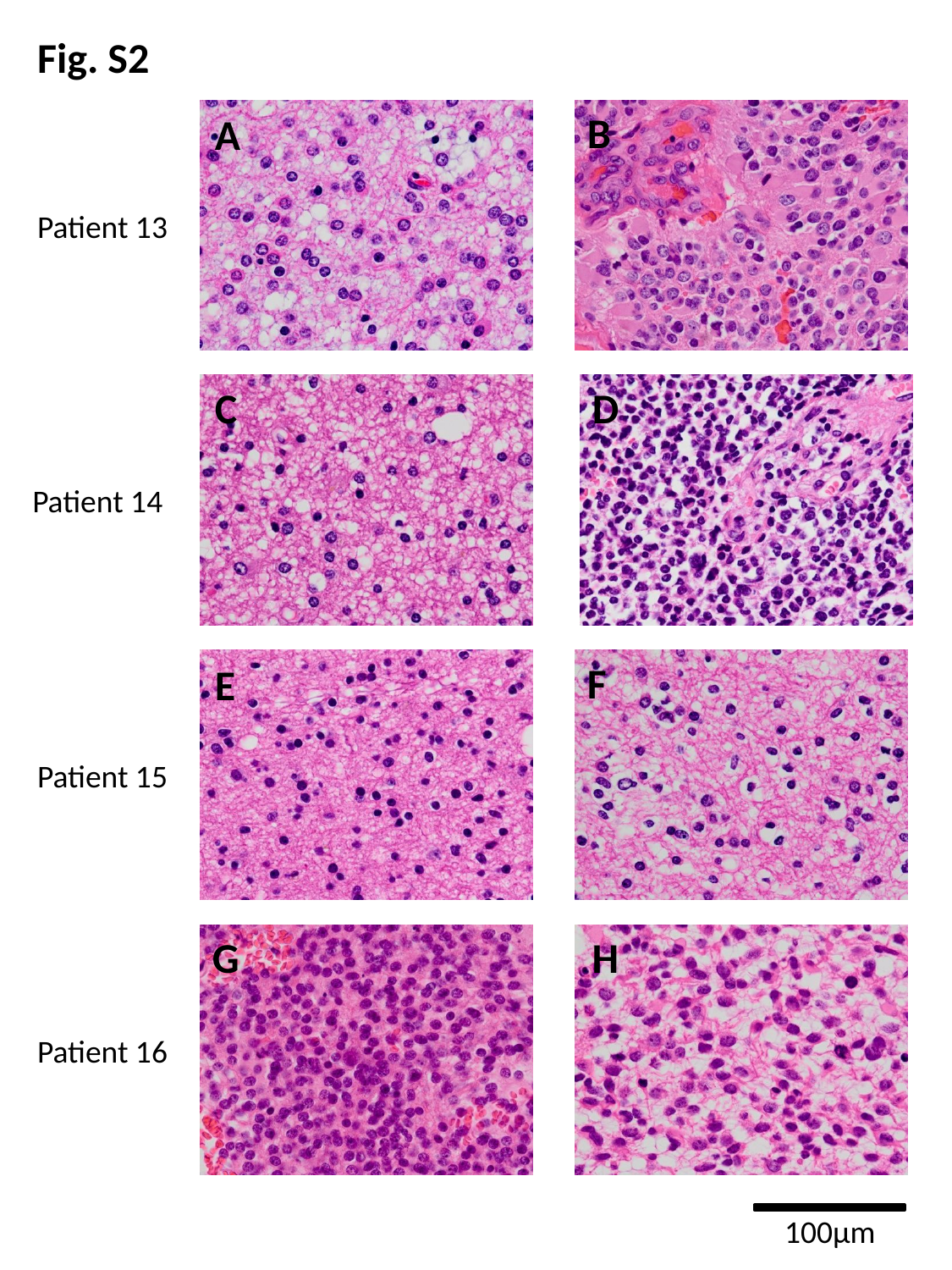

Fig. S2
B
A
Patient 13
C
D
Patient 14
F
E
Patient 15
H
G
Patient 16
100μm
